# Growth factor genes and change in mammographic density after stopping combined hormone therapy in the California Teachers Study

**DOI:** 10.1186/s12885-018-4981-6

**Published:** 2018-11-06

**Authors:** Eunjung Lee, Jianning Luo, Fredrick R. Schumacher, David Van Den Berg, Anna H. Wu, Daniel O. Stram, Leslie Bernstein, Giske Ursin

**Affiliations:** 1 Department of Preventive Medicine, Keck School of Medicine, University of Southern California/Norris Comprehensive Cancer Center, Los Angeles, CA 90089 USA; 2 Department of Population Sciences, Beckman Research Institute, City of Hope, Duarte, CA 91010 USA; 3 Department of Population and Quantitative Health Sciences, Case Western Reserve University, Cleveland, OH 44106 USA; 4 Department of Nutrition, University of Oslo, Oslo, Norway; 5 Cancer Registry of Norway, Oslo, Norway

**Keywords:** Hormone therapy, Mammographic density, Polymorphisms, Growth factor pathway

## Abstract

**Background:**

The contribution of genetic polymorphisms to the large inter-individual variation in mammographic density (MD) changes following starting and stopping use of estrogen and progestin combined therapy (EPT) has not been well-studied. Previous studies have shown that circulating levels of insulin-like growth factors are associated with MD and cross-talk between estrogen signaling and growth factors is necessary for cell proliferation in the breast. We evaluated single nucleotide polymorphisms (SNPs) in growth factor genes in association with MD changes after women stop EPT use.

**Methods:**

We genotyped 191 SNPs in 13 growth factor pathway genes in 284 non-Hispanic white California Teachers Study participants who previously used EPT and collected their mammograms before and after quitting EPT. Percent MD was assessed using a computer-assisted method. Change in percent MD was calculated by subtracting percent MD of an ‘off-EPT’ mammogram from percent MD of an ‘on-EPT’ (i.e. baseline) mammogram. We used multivariable linear regression analysis to investigate the association between SNPs and change in percent MD. We calculated *P*-values corrected for multiple testing within a gene (P_adj_).

**Results:**

Rs1983210 in *INHA* and rs35539615 in *IGFBP1/3* showed the strongest associations. Per minor allele of rs1983210, the absolute change in percent MD after stopping EPT use decreased by 1.80% (a difference in absolute change in percent MD) (P_adj_= 0.021). For rs35539615, change in percent MD increased by 1.79% per minor allele (P_adj_= 0.042). However, after applying a Bonferroni correction for the number of genes tested, these associations were no longer statistically significant.

**Conclusions:**

Genetic variation in growth factor pathway genes *INHA* and *IGFBP1/3* may predict longitudinal MD change after women quit EPT. The observed differences in EPT-associated changes in percent MD in association with these genetic polymorphisms are modest but may be clinically significant considering that the magnitude of absolute increase in percent MD reported from large clinical trials of EPT ranged from 3% to 7%.

**Electronic supplementary material:**

The online version of this article (10.1186/s12885-018-4981-6) contains supplementary material, which is available to authorized users.

## Introduction

Mammographic density (MD), one of the strongest risk factors for breast cancer, is a measure of the amount of epithelium and stroma in the breast [1]. Estrogen and progestin combined therapy (EPT) use was shown to increase MD in two randomized clinical trials, the Postmenopausal Estrogen/Progestin Interventions (PEPI) trial and the Women’s Health Initiative (WHI) randomized trial. In these trials, women randomized to take EPT experienced an average increase of 3-7% in MD [2–4]. Similarly, MD decreased after discontinuing EPT [5, 6]. In a randomized trial, EPT users who stopped their hormone use for 1 month and 2 months showed reductions in MD of 0.9% and 1.5%, respectively, whereas no reduction was observed among women who continued using EPT [6]. However, inter-individual variation in MD changes was large following EPT use [2–4] or cessation [6, 7]. For example, among women in the EPT arm of the PEPI trial, ~20% experienced a large increase (i.e. a one-category increase in Breast Imaging Reporting and Data System (BI-RADS) grade, corresponding to ~14-18% increase in MD), while other women experienced smaller changes [2, 3]. Among women in the cessation group of the short-term cessation trial, about 25% experienced at least a 7.5% decrease in MD while ~ 55% experienced a modest decrease or little change and ~ 20% experienced some increase after EPT cessation [7].

The evidence showing that breast cancer risk increases or decreases in accordance with longitudinal changes in MD is consistent [8–10]. Importantly, the longitudinal change in MD after using EPT explained all of the increased risk from EPT use in the WHI [9]. However, despite the large inter-individual variation in MD change when starting EPT and quitting EPT [2–4, 6, 7], determinants of EPT-associated MD change remain largely unknown. In the short-term cessation trial, none of the tested risk factors for breast cancer, such as race, BMI, parity, family history, and duration of hormone therapy use, appeared to determine the amount of MD decrease following hormone cessation [7], suggesting that genetic factors might be important determinants [7]. A similar conclusion was derived from the results of a study of MD increase following initiation of hormone therapy that was predominantly EPT [11]. Only a few studies, all focusing on hormone metabolism pathway, have investigated genetic determinants of MD change associated with EPT use or cessation [12–15]. The most comprehensive study to date, our investigation of 30 hormone metabolism pathway genes using data from the California Teachers Study (CTS) mammogram substudy, showed that SNPs in *SLCO1B1* (rs7489119) and *ARSC* (rs5933863) to be associated with MD decrease following EPT cessation [12]. Other SNPs reported from smaller studies were not replicated [13–15].

Along with the female sex steroid hormone signaling cascade, paracrine signals by growth factors such as epidermal growth factor (EGF) and insulin-like growth factor-1 (IGF-1) are crucial in mammary tissue development [16, 17]. Although results from studies of growth factor polymorphisms and MD have been inconsistent [18–21], several cross-sectional studies have shown that circulating or breast tissue levels of IGFs are associated with MD [22–26]. Further, experimental evidence in breast cancer cell lines suggests that cross-talk between signaling pathways of steroids and growth factors such as EGF or IGF-1 is necessary to induce cell growth and proliferation [17]. Thus, we hypothesized that polymorphisms in growth factor pathway genes may contribute to the inter-individual differences in EPT-associated MD change and investigated associations between 191 SNPs in 13 growth factor genes and change in MD with cessation of EPT use in the mammogram substudy of the CTS.

## Materials and Methods

### Participants

The CTS mammogram substudy was established to identify genetic polymorphisms associated with MD changes when women initiate or quit EPT use. The design of this substudy was previously described [12]. Briefly, the CTS is a prospective cohort established in 1995-1996 and consists of 133,479 current or former female public school teachers and other public school professionals, who were members of the California State Teachers Retirement System in 1995. When the cohort was formed, participants completed and returned a mailed questionnaire which included questions on menstrual history, parity, hormone use, and medical history [27].

In 2006-2008, we mailed study invitation letters to 1,420 CTS participants who lived in California, did not have a cancer diagnosis, were ages 40-60 years, had a mammogram in the 2 years prior to cohort enrollment, initiated EPT use between cohort enrollment and completion of the follow-up questionnaire in 2000-2001, and did not participate in any other CTS substudy. We telephoned 1,272 women and identified 1,251 eligible women. A total of 1,004 eligible women participated and completed a telephone interview providing current information on menstrual history and hormone use. Reasons for non-participation included refusal (*n* = 111), not returning signed informed consent (*n* = 134), withdrawing consent (*n* = 1), and not completing the interview (*n* = 1). We collected mammograms for 993 women, but excluded 29 who were missing information on hormone therapy use. On average, 8 mammograms for each participant were available. About 40% of these mammograms were sent with a form summarizing history of menstruation and hormone use as of the day of screening. This information is expected to be more accurate than the information collected during the interview. Thus, we used information from the records when available [12]. The CTS mammographic density substudy was approved by the University of Southern California (USC) Institutional Review Board (HS-056027). All participants provided written informed consent.

### Identifying EPT quitters

Detailed methods to select mammograms and identify EPT quitters have been described [12]. Briefly, to determine MD changes following the changes in women’s EPT use, we selected an “on-EPT mammogram” (taken while a participant was post-menopausal and using EPT) and an “off-EPT mammogram” (taken while a participant was postmenopausal and not using EPT or ET). Because many participants could not recall exactly when they started or stopped hormone therapy [12] and many EPT users quit hormones after the publication of WHI findings in June 2002 [28], we preferred that on-EPT mammograms were taken before July 1, 2002, and off-EPT mammograms were taken after 2003 to minimize misclassification of EPT use information at time of mammograms [12]. When selecting off-EPT mammograms, we preferred mammograms taken as close as possible to the year of on-EPT mammograms, but with at least one year interval between the on-EPT and off-EPT mammograms; this was important because MD changes after starting EPT use were reported to occur primarily within the first 12 months and remain constant for at least the next two years [2].

Applying these criteria, we selected both an “on-EPT mammogram” and an “off-EPT mammogram” for 422 women. The majority (*n* = 371) were ‘quitters’ as opposed to ‘starters’ (*n* = 51) in that the time sequence of their mammograms was an “on-EPT mammogram” followed by an “off-EPT mammogram”. When inspecting the year of menopause and year of EPT initiation, we were concerned about the possibility that the “off-EPT mammograms” we collected from the ‘starters’ were in fact taken during their menopausal transition. Because MD change we measured on these starters is represented by a mixture of a decline in MD associated with menopausal transition (i.e. hormone levels decrease [29, 30]) and an increase in MD associated with initiation of EPT after they became completely postmenopausal (i.e. hormone levels increase), we restricted the study to the 371 EPT quitters [12].

### MD assessment

Methods for MD measurements have been described [3, 31]. Mammograms were digitized using a Cobrascan CX812T scanner (Radiographic Digital Imaging, Torrance, CA) at a resolution of 150 pixels/inch (59 dots/cm). One of the authors (GU) assessed absolute density of digitized mammograms using the USC Madena method, which is a validated, computer-assisted, quantitative technique [32]. One research assistant trained by GU assessed total area of the breast. MD (in percent) was calculated as the absolute density divided by the total area of the breast. Mammograms of the same individual were evaluated in the same batch. Readers were blinded to subject identification and EPT use status for each mammogram. Reader reproducibility based on 183 pairs of random blinded duplicates was excellent (R=0.96).

### Specimen collection and genotyping

Spit samples were collected using the Oragene DNA self-collection kit (DNA Genotek, Kanata, ON, Canada) which was mailed to participants with a return package. Of the 371 EPT quitters, 328 women provided a sufficient amount of sample. Linkage disequilibrium (LD) tagging SNPs were selected across each gene, from 20kb upstream of 5′ untranslated region (UTR) and to 10kb downstream of 3′ UTR, using the Snagger software (for *FGF2, FGF9, IGF1, IGFBP1/3, RPS6KA1, TGFB1*) [33] and the TagSNPs program (for *ACVR1, ACVR2, IGF2, INHA, INHBA, INHBB*) [34, 35]. The selected SNPs were to tag all common SNPs (minor allele frequency (MAF) ≥ 5%) in whites with minimum pairwise r^2^ of 0.80. For *EGFR*, we selected a few SNPs due to limited space in the genotyping platform [12].

Genotyping was performed in the USC Core Facility using the Illumina GoldenGate Assay (Illumina, San Diego, CA, USA). After excluding SNPs with a call rate <90% (15 SNPs), Hardy-Weinberg equilibrium *P*-value <0.001 (2 SNPs), and MAF<1% (2 SNPs), 191 SNPs remained for analyses. The number of SNPs genotyped for each gene is listed in Table 1. After excluding samples with a call rate <90% (*n* = 19), 309 samples were available for analyses. The duplicate genotyping concordance was >99.9% [12].

**Table 1:** List of investigated growth factor pathway genes and number of genotyped single nucleotide polymorphisms (SNPs)

Gene	Gene name	N of SNPs genotyped
*EGFR*	Epidermal growth factor receptor	7
*FGF2*	Fibroblast growth factor 2	24
*FGF9*	Fibroblast growth factor 9	15
*IGF1*	Insulin like growth factor 1	18
*IGF2*	Insulin like growth factor 2	7
*IGFBP1/3*	Insulin like growth factor binding protein 1/3	24
*RPS6KA1*	Ribosomal protein S6 kinase A1	19
*INHA*	Inhibin alpha subunit	12
*INHBA*	Inhibin beta A subunit	15
*INHBB*	Inhibin beta B subunit	15
*TGFB1*	Transforming growth factor beta 1	7
*ACVR1*	Activin A receptor type 1	20
*ACVR2*	Activin A receptor type 2	9

Because the majority (*n* = 284) of these remaining 309 samples were from participants who were non-Hispanic white and the number of participants in other race/ethnic groups was limited, we restricted the analysis to 284 non-Hispanic whites. Among the 284 women genotyped and eligible for the analyses, the time interval between the on-EPT mammogram and off-EPT mammogram was 5 years or less for over 85% of the participants [12]. Applying the methods to calculate MD change described above, the mean change in percent MD was 4.0% (± 7.0% SD) among the participants included in the analyses [12]. Of the 422 women for whom we collected both on-EPT mammograms and off-EPT mammograms, the 284 non-Hispanic white women included in the analysis differed from the excluded (*n* = 138) women with respect to time interval between the two mammograms and age at time of off-EPT mammogram, reflecting the inclusion criteria (i.e. non-Hispanic white, EPT ‘quitters’ as opposed ‘starters’). The two groups were similar with respect to age, BMI, parity, menopausal status, history of breast biopsy, and family history of breast cancer (Additional file 1: Table S1).

### Statistical Analysis

We calculated change in percent MD as “on-EPT MD” minus “off-EPT MD”, representing the absolute change in percent MD after stopping EPT use. Multivariable linear regression analysis was used to evaluate the association between genotype and change in percent MD, adjusting for age and BMI at baseline (i.e. on-EPT mammogram), time interval and BMI change between the two mammograms, and baseline MD (i.e. “on-EPT MD”). Parity was not associated with change in MD and was not included in the model; additional adjustment for parity did not change the results (data not presented). We used an additive genetic model, which estimates the difference in change in percent MD per minor allele. Thus, the regression coefficient from this model indicates the absolute difference, per minor allele of the modeled SNP, in absolute change in percent MD after stopping EPT. Compared to cross-sectional MD ranging from 0% to 100%, which shows a highly skewed distribution and requires square-root transformation to improve normality of residuals in regression models [36], longitudinal change in percent MD has a distribution more closely approximate a normal distribution. We calculated *P*-values corrected for multiple correlated tests within each gene using a method which considers correlation between SNPs (P_adj_) [37].

We also performed exploratory analyses stratified by parity (nulliparous, parous) and calculated P values for interaction by conducting Wald tests for genotype-parity product terms. This was planned a *priori* because nulliparity is a risk factor of breast cancer and higher MD [38, 39], and MD was more strongly associated with breast cancer risk in nulliparous women than in parous women [40].

## Results

We observed statistically significant associations for rs1983210 in *INHA* and rs35539615 in *IGFBP1/3* region after we corrected for multiple testing in each gene. The longitudinal absolute change in percent MD after stopping EPT decreased by 1.80% (an absolute difference, not a relative difference, in absolute change in percent MD after stopping EPT) per minor allele of rs1983210 (*INHA*; P_adj_=0.021) and increased by 1.79% per minor allele of rs35539615 (*IGFBP1/3;* P_adj_=0.042; Table 2). However, if we further consider all tested genes and apply multiple testing correction for all SNPs tested, neither of these associations remained statistically significant. Another SNP in *INHA* (rs2278200) showed some evidence of association with borderline statistical significance (P_adj_=0.053), but this association was not observed when adjusting for rs1983210. Results for all tested SNPs are presented in Additional file 2: Table S2.

**Table 2:** Single nucleotide polymorphisms (SNPs) associated with estrogen and progestin combined therapy (EPT)-associated mammographic density change after multiple testing correction at gene level^a^

SNP (gene)	Major/minor allele	Minor allele frequency	N (WW/WV/VV)	Beta (SE)	P (P_adj_^b^)
rs1983210 (*INHA*)	G/C	0.28	156/104/21	-1.80 (0.59)	0.003 (0.021)
rs35539615 (*IGFBP1/3*)	C/G	0.26	160/104/20	1.79 (0.59)	0.002 (0.043)
rs2278200 (*INHA*)^c^	C/G	0.45	70/137/53	-1.55 (0.57)	0.008 (0.053)

^a^Based on linear regression models adjusting for age and body mass index (BMI) at time of on-EPT mammogram, time interval and BMI change between the two mammograms, and mammographic density of on-EPT mammogram. Additive genetic model was used

^b^*P*-values adjusted for multiple correlated tests (P_ACT_) [37]

^c^rs1983210 and rs2278200 (*INHA*), r^2^=0.43

When examining the associations separately in nulliparous vs. parous women, four SNPs (rs2278200, rs907142, rs2059693, rs1039898) in *INHA* were associated with change in percent MD in nulliparous women (P_adj_<0.05; P values for interaction <0.022; Table 3), and the regression coefficients for these SNPs from the nulliparous women analyses (3.71% to 5.16% absolute difference in change in percent MD per minor allele; Table 3) were much larger than the coefficients for the most statistically significant SNPs from the overall analyses (1.55% to 1.80% absolute difference per minor allele; Table 2). Rs4674413 in *INHA* was also associated with change in percent MD, but this SNP was in LD with rs2059693 (r^2^=0.98). None of these or other tested SNPs showed statistically significant associations in parous women.

**Table 3:** Single nucleotide polymorphisms (SNPs) associated with estrogen and progestin combined therapy (EPT)-associated mammographic density change among nulliparous women after multiple testing correction at gene level; results from parous women (*n* = 219) are presented for comparison^a^

SNP (gene)	Major/minor allele	Minor allele frequency	Nulliparous (*n* = 63)				Parous (*n* = 219)				P for interaction^c^
			N (WW/WV/VV)	Beta (SE)	P	P_adj_^b^	N (WW/WV/VV)	Beta (SE)	P	P_adj_^b^	
rs2278200^d^ (*INHA*)	C/G	0.45	17/23/15	-3.78 (1.03)	0.0006	0.005	51/114/38	-0.49 (0.68)	0.47	>0.99	0.013
rs907142^d^(*INHA*)	G/C	0.32	27/30/4	3.97 (1.31)	0.004	0.028	82/101/20	0.21 (0.68)	0.76	>0.99	0.007
rs2059693^d^ (*INHA*)	C/T	0.28	31/27/4	3.71 (1.27)	0.005	0.033	107/94/16	0.10 (0.68)	0.88	>0.99	0.013
rs1039898 (*INHA*)	T/C	0.10	50/13/0	5.16 (1.91)	0.009	0.048	183/34/1	-0.14 (1.09)	0.90	>0.99	0.022

^a^Based on linear regression model adjusting for age and body mass index (BMI) at time of on-EPT mammogram, time interval and BMI change between the two mammograms, and mammographic density of on-EPT mammogram. Additive genetic model was used

^b^Multiple testing corrected *P*-value; P_ACT_ (P values adjusted for correlated tests) within each gene was calculated using the methods by Conneely and Boehnke [35]

^c^*P*-values for interaction were not corrected for multiple testing

^d^rs2278200 and rs907142, r^2^=0.37; rs907142 and rs2059693, r^2^=0.55

## Discussion

In this longitudinal study, two SNPs located near growth factor pathway genes *INHA* and *IGFBP1/3* were associated with change in percent MD after quitting EPT after we corrected for multiple testing in each gene. To our knowledge, this is the first investigation of growth factor pathway genes in relation to longitudinal change in percent MD after EPT cessation. Our findings will contribute to improving our understanding of breast cancer risk in current and former EPT users as well as in women who are considering EPT use for menopausal symptoms.

Experimental evidence in breast cancer and other cell lines suggests that steroids and growth factor signaling pathways such as EGF and IGF-1 have synergistic effects in inducing cell proliferation and that cross-talk between these pathways is important in estrogen action [41–45].

Rs1983210, which showed an association with the smallest *P*-value, is located about 15kb upstream of the *INHA* gene in chromosome 2. *INHA* encodes the inhibin α, a subunit of heterodimeric glycoproteins called inhibins. Inhibins are members of the TGF-β superfamily, are expressed in normal human breast tissue [46, 47] and are involved in multiple aspects of cell differentiation and proliferation including mammary gland development [47, 48]. While the functional significance of rs1983210 on the expression of inhibin α is not known, results from a microarray-based expression profiling study have shown that lower expression of TGF-β pathway genes was associated with higher MD [49], supporting the role of *INHA* SNPs in determining EPT-associated MD change. Alternatively, our observation may be related to the amino acid change (Glu1365Asp) in *obscurin like 1* (*OBSL1*) gene resulting from rs1983210. Although rs1983210 was included in our study as a part of the SNPs located in the vicinity (i.e. from 20kb upstream to 10kb downstream) of *INHA*, rs1983210 is in fact located in exon 2 of *OBSL1* and leads to amino acid change (Glu1365Asp). While the function of *OBSL1* gene is not well characterized, rare mutations in *OBSL1* lead to severe pre-natal and postnatal growth restriction known as 3-M syndrome, an autosomal-recessive growth disorder [50–52]. In one experimental study, a fibroblast cell line with an *OBSL1* mutation displayed disrupted IGF-1 signaling [50]. Rs1983210 was assessed as not having clinical significance in relation to 3-M disease in dbSNP [53], but it is not known whether this SNP might contribute to other common diseases. The observed association for rs1983210 should be replicated in future studies and it needs to be determined whether the association is related to *INHA* or *OBSL1*.

*IGFBP1* and *IGFBP3* genes are located adjacent to each other and are important regulators of bioavailability of IGF-1, which stimulates proliferation of breast cancer cell lines and primary culture [43, 45]. Cross-sectional studies investigating polymorphisms in *IGF1* and *IGFBP1/3* in association with MD have reported mixed results [18–21, 54, 55]. However, several lines of evidence indicate that our observation for rs35539615 in relation to EPT-associated MD change is biologically plausible. Cross-sectional studies have shown that higher IGF-1 levels and lower IGFBP-3 levels in blood samples are associated with higher MD [23–25, 56, 57]. In all [23–25, 56] but one [57] of these studies, the associations were observed among premenopausal women but not among postmenopausal women who were not taking hormone therapy, supporting the role of the interaction between IGF-1/IGFBP-3 and female sex steroid hormones in determining MD. Data from experimental and animal studies also show that IGF-1 and estrogen signaling pathways activate each other and that IGF-1, estrogen, and progesterone synergistically stimulate mammary gland development [42, 58, 59]. Further, in a small phase I clinical trial, decreases in cell proliferation in atypical hyperplasia and proliferative lesions in the breast were observed in patients diagnosed with atypical hyperplasia and treated with an IGF-1 pathway inhibitor, pasireotide, but not in untreated patients [60].

It is worth noting that estrogen therapy reduces circulating levels of IGF-1 and possibly IGFBP-3 and increases IGFBP-1 levels [61]. Addition of androgenic progestins such as medroxyprogesterone acetate or norethisterone appears to oppose the estrogen therapy effect on IGF and IGFBP levels [61, 62]. Nevertheless, future studies of IGF pathway polymorphisms should examine and consider any changes in IGF and IGFBP levels associated with EPT use.

These results suggest that genetic variation in *INHA* and *IGFBP1/3* may influence change in percent MD following EPT cessation. The estimated absolute differences of 1.80% (for rs1983210) and 1.79% (for rs35539615) in change in percent MD per minor allele are modest; however, when considering that the absolute increases in percent MD following EPT treatment in the WHI trial and the PEPI trial were 3-7% on average [2–4] and that each 1% increase in MD (absolute increase in percent MD) increased breast cancer risk by 3% [9], these observed differences may be clinically significant. Further, our exploratory investigation stratifying by parity indicates that SNPs in *INHA* may have greater influence in nulliparous women. The larger regression coefficients for SNPs in the analysis of nulliparous women compared to those in the analysis of parous women suggest that genetic variation may be more important for nulliparous women as determinants of the EPT-associated change in percent MD. This is a potentially important observation considering that nulliparous women have greater breast cancer risk than parous women [63] and that identifying risk factors for nulliparous women is important. Breast tissue of nulliparous women may differ biologically from that of parous women and this may be associated with different gene expression patterns [63, 64]. Future investigations need to include a sufficient number of nulliparous women to attain adequate statistical power.

To our knowledge, this is the first investigation of growth factor pathway as genetic predictors of EPT-associated changes in percent MD. Additional strengths include that MD of all mammograms was estimated by one experienced investigator using a validated method and each pair of the before- and after-quitting mammograms was assessed in the same batch. One limitation, which our group acknowledged previously [12], is that our study did not include a comparison group whose EPT use status did not change between two mammograms, such as those who continued to use EPT or who never used EPT. Thus, it is possible that the identified genetic predictors of EPT-associated change in percent MD might also be associated with natural decreases in percent MD due to aging. Additional limitation is that this study was conducted in non-Hispanic whites only. Future investigations in other race/ethnic groups are warranted especially for SNPs with higher MAFs in other race/ethnic groups such as African Americans. Our observations, if confirmed in larger studies that include a comparison group, will be clinically important since some women still use EPT for their menopausal symptoms, and if we can identify genetic predictors of the decrease in percent MD after quitting EPT, this information will help women and physicians to make informed decisions when they consider using EPT.

## Conclusions

In this longitudinal study, polymorphisms in growth factor pathway genes *INHA* and *IGFBP1/3* were associated with changes in percent MD after women quit EPT. If confirmed in larger studies as well as in women starting EPT, our findings may help to identify a subgroup of women who will benefit from EPT with minimal increase in breast cancer risk and a subgroup of women who will be subject to a large increase in risk associated with EPT and thus should avoid starting EPT at all.

## Additional files

Additional file 1: **Table S1.** Comparison of characteristics of women who were included in the analyses with characteristics of participants who were included in the longitudinal set (i.e. both on-EPT and off-EPT mammograms were available) but excluded from the analyses. (DOCX 14 kb)

Additional file 2: **Table S2.** Association between mammographic density change after quitting EPT use and SNPs in growth factor genes. (XLSX 47 kb)

## Abbreviations

BI-RADS: Breast Imaging Reporting and Data System
BMI: Body mass index;
CEE: Conjugated equine estrogens
CTS: California Teachers Study
EPIC: European Prospective Investigation into Cancer and Nutrition
EPT: Estrogen and progestin combined therapy
MD: Mammographic density
PEPI: Postmenopausal Estrogen/Progestin Interventions
SNP: Single nucleotide polymorphism
WHI: Women’s Health Initiative

## References

[1] McCormack VA, dos Santos Silva I (2006). Breast density and parenchymal patterns as markers of breast cancer risk: a meta-analysis. Cancer Epidemiol Biomarkers Prev..

[2] Greendale GA, Reboussin BA, Sie A, Singh HR, Olson LK, Gatewood O (1999). Effects of estrogen and estrogen-progestin on mammographic parenchymal density. Postmenopausal Estrogen/Progestin Interventions (PEPI) Investigators. Ann Intern Med..

[3] Greendale GA, Reboussin BA, Slone S, Wasilauskas C, Pike MC, Ursin G (2003). Postmenopausal hormone therapy and change in mammographic density. J Natl Cancer Inst..

[4] McTiernan A, Martin CF, Peck JD, Aragaki AK, Chlebowski RT, Pisano ED (2005). Estrogen-plus-progestin use and mammographic density in postmenopausal women: Women's Health Initiative randomized trial. J Natl Cancer Inst..

[5] Rutter CM, Mandelson MT, Laya MB, Seger DJ, Taplin S (2001). Changes in breast density associated with initiation, discontinuation, and continuing use of hormone replacement therapy. JAMA..

[6] Buist DS, Anderson ML, Reed SD, Aiello Bowles EJ, Fitzgibbons ED, Gandara JC (2009). Short-term hormone therapy suspension and mammography recall: a randomized trial. Ann Intern Med..

[7] Lowry SJ, Aiello Bowles EJ, Anderson ML, Buist DS (2011). Predictors of breast density change after hormone therapy cessation: results from a randomized trial. Cancer Epidemiol Biomarkers Prev..

[8] Kerlikowske K, Ichikawa L, Miglioretti DL, Buist DS, Vacek PM, Smith-Bindman R (2007). Longitudinal measurement of clinical mammographic breast density to improve estimation of breast cancer risk. J Natl Cancer Inst..

[9] Byrne C, Ursin G, Martin CF, Peck JD, Cole EB, Zeng D et al. Mammographic Density Change With Estrogen and Progestin Therapy and Breast Cancer Risk. J Natl Cancer Inst. 2017;109(9):djx001. 10.1093/jnci/djx001 PMC6059178 28376149

[10] Cuzick J, Warwick J, Pinney E, Duffy SW, Cawthorn S, Howell A (2011). Tamoxifen-induced reduction in mammographic density and breast cancer risk reduction: a nested case-control study. J Natl Cancer Inst..

[11] Vachon CM, Sellers TA, Vierkant RA, Wu FF, Brandt KR (2002). Case-control study of increased mammographic breast density response to hormone replacement therapy. Cancer Epidemiol Biomarkers Prev..

[12] Lee E, Luo J, Su YC, Lewinger JP, Schumacher FR, Van Den Berg D (2014). Hormone metabolism pathway genes and mammographic density change after quitting estrogen and progestin combined hormone therapy in the California Teachers Study. Breast Cancer Res..

[13] Lord SJ, Mack WJ, Van Den Berg D, Pike MC, Ingles SA, Haiman CA (2005). Polymorphisms in genes involved in estrogen and progesterone metabolism and mammographic density changes in women randomized to postmenopausal hormone therapy: results from a pilot study. Breast Cancer Res..

[14] van Duijnhoven FJ, Peeters PH, Warren RM, Bingham SA, Uitterlinden AG, van Noord PA (2006). Influence of estrogen receptor alpha and progesterone receptor polymorphisms on the effects of hormone therapy on mammographic density. Cancer Epidemiol Biomarkers Prev..

[15] Lee E, Ingles SA, Van Den Berg D, Wang W, Lavallee C, Huang MH (2012). Progestogen levels, progesterone receptor gene polymorphisms, and mammographic density changes: results from the Postmenopausal Estrogen/Progestin Interventions Mammographic Density Study. Menopause..

[16] Sternlicht MD (2006). Key stages in mammary gland development: the cues that regulate ductal branching morphogenesis. Breast Cancer Res..

[17] Fox EM, Andrade J, Shupnik MA (2009). Novel actions of estrogen to promote proliferation: integration of cytoplasmic and nuclear pathways. Steroids..

[18] Tamimi RM, Cox DG, Kraft P, Pollak MN, Haiman CA, Cheng I (2007). Common genetic variation in IGF1, IGFBP-1, and IGFBP-3 in relation to mammographic density: a cross-sectional study. Breast Cancer Res..

[19] Verheus M, Maskarinec G, Woolcott CG, Haiman CA, Le Marchand L, Henderson BE (2010). IGF1, IGFBP1, and IGFBP3 genes and mammographic density: the Multiethnic Cohort. Int J Cancer..

[20] Ozhand A, Lee E, Wu AH, Ellingjord-Dale M, Akslen LA, McKean-Cowdin R (2013). Variation in inflammatory cytokine/growth-factor genes and mammographic density in premenopausal women aged 50-55. PloS one..

[21] Ellingjord-Dale M, Lee E, Couto E, Ozhand A, Qureshi SA, Hofvind S (2012). Polymorphisms in hormone metabolism and growth factor genes and mammographic density in Norwegian postmenopausal hormone therapy users and non-users. Breast Cancer Res..

[22] Guo YP, Martin LJ, Hanna W, Banerjee D, Miller N, Fishell E (2001). Growth factors and stromal matrix proteins associated with mammographic densities. Cancer Epidemiol Biomarkers Prev..

[23] dos Santos SI, Johnson N, De Stavola B, Torres-Mejia G, Fletcher O, Allen DS (2006). The insulin-like growth factor system and mammographic features in premenopausal and postmenopausal women. Cancer Epidemiol Biomarkers Prev..

[24] Diorio C, Pollak M, Byrne C, Masse B, Hebert-Croteau N, Yaffe M (2005). Insulin-like growth factor-I, IGF-binding protein-3, and mammographic breast density. Cancer Epidemiol Biomarkers Prev..

[25] Byrne C, Colditz GA, Willett WC, Speizer FE, Pollak M, Hankinson SE (2000). Plasma insulin-like growth factor (IGF) I, IGF-binding protein 3, and mammographic density. Cancer Res..

[26] Boyd NF, Stone J, Martin LJ, Jong R, Fishell E, Yaffe M (2002). The association of breast mitogens with mammographic densities. Br J Cancer..

[27] Bernstein L, Allen M, Anton-Culver H, Deapen D, Horn-Ross PL, Peel D (2002). High breast cancer incidence rates among California teachers: results from the California Teachers Study (United States). Cancer Causes Control..

[28] Marshall SF, Clarke CA, Deapen D, Henderson K, Largent J, Neuhausen SL (2010). Recent breast cancer incidence trends according to hormone therapy use: the California Teachers Study cohort. Breast Cancer Res..

[29] Rannevik G, Jeppsson S, Johnell O, Bjerre B, Laurell-Borulf Y, Svanberg L (1995). A longitudinal study of the perimenopausal transition: altered profiles of steroid and pituitary hormones, SHBG and bone mineral density. Maturitas..

[30] Longcope C, Franz C, Morello C, Baker R, Johnston CC (1986). Steroid and gonadotropin levels in women during the peri-menopausal years. Maturitas..

[31] Ursin G, Palla SL, Reboussin BA, Slone S, Wasilauskas C, Pike MC (2004). Post-treatment change in serum estrone predicts mammographic percent density changes in women who received combination estrogen and progestin in the Postmenopausal Estrogen/Progestin Interventions (PEPI) Trial. J Clin Oncol..

[32] Ursin G, Astrahan MA, Salane M, Parisky YR, Pearce JG, Daniels JR (1998). The detection of changes in mammographic densities. Cancer Epidemiol Biomarkers Prev..

[33] Edlund CK, Lee WH, Li D, Van Den Berg DJ, Conti DV (2008). Snagger: a user-friendly program for incorporating additional information for tagSNP selection. BMC Bioinformatics..

[34] Canzian F, Cox DG, Setiawan VW, Stram DO, Ziegler RG, Dossus L (2010). Comprehensive analysis of common genetic variation in 61 genes related to steroid hormone and insulin-like growth factor-I metabolism and breast cancer risk in the NCI breast and prostate cancer cohort consortium. Hum Mol Genet..

[35] Stram DO, Haiman CA, Hirschhorn JN, Altshuler D, Kolonel LN, Henderson BE (2003). Choosing haplotype-tagging SNPS based on unphased genotype data using a preliminary sample of unrelated subjects with an example from the Multiethnic Cohort Study. Hum Hered..

[36] Burton A, Maskarinec G, Perez-Gomez B, Vachon C, Miao H, Lajous M (2017). Mammographic density and ageing: A collaborative pooled analysis of cross-sectional data from 22 countries worldwide. PLoS Med..

[37] Conneely KN, Boehnke M (2007). So Many Correlated Tests, So Little Time! Rapid Adjustment of P Values for Multiple Correlated Tests. Am J Hum Genet..

[38] Vachon CM, Kuni CC, Anderson K, Anderson VE, Sellers TA (2000). Association of mammographically defined percent breast density with epidemiologic risk factors for breast cancer (United States). Cancer Causes Control..

[39] Stone J, Warren RM, Pinney E, Warwick J, Cuzick J (2009). Determinants of percentage and area measures of mammographic density. Am J Epidemiol..

[40] Woolcott CG, Koga K, Conroy SM, Byrne C, Nagata C, Ursin G (2012). Mammographic density, parity and age at first birth, and risk of breast cancer: an analysis of four case-control studies. Breast Cancer Res Treat..

[41] Skandalis SS, Afratis N, Smirlaki G, Nikitovic D, Theocharis AD, Tzanakakis GN (2014). Cross-talk between estradiol receptor and EGFR/IGF-IR signaling pathways in estrogen-responsive breast cancers: focus on the role and impact of proteoglycans. Matrix Biol.

[42] Dupont J, Karas M, LeRoith D (2000). The potentiation of estrogen on insulin-like growth factor I action in MCF-7 human breast cancer cells includes cell cycle components. J Biol Chem..

[43] Hawsawi Y, El-Gendy R, Twelves C, Speirs V, Beattie J (1836). Insulin-like growth factor - oestradiol crosstalk and mammary gland tumourigenesis. Biochim Biophys Acta..

[44] Molloy CA, May FE, Westley BR (2000). Insulin receptor substrate-1 expression is regulated by estrogen in the MCF-7 human breast cancer cell line. J Biol Chem..

[45] Westley BR, May FE (1994). Role of insulin-like growth factors in steroid modulated proliferation. J Steroid Biochem Mol Biol..

[46] Di Loreto C, Reis FM, Cataldi P, Zuiani C, Luisi S, Beltrami CA (1999). Human mammary gland and breast carcinoma contain immunoreactive inhibin/activin subunits: evidence for a secretion into cystic fluid. Eur J Endocrinol..

[47] Reis FM, Luisi S, Carneiro MM, Cobellis L, Federico M, Camargos AF (2004). Activin, inhibin and the human breast. Mol Cellular Endocrinol..

[48] Burdette JE, Jeruss JS, Kurley SJ, Lee EJ, Woodruff TK (2005). Activin A mediates growth inhibition and cell cycle arrest through Smads in human breast cancer cells. Cancer Res..

[49] Yang WT, Lewis MT, Hess K, Wong H, Tsimelzon A, Karadag N (2010). Decreased TGFbeta signaling and increased COX2 expression in high risk women with increased mammographic breast density. Breast Cancer Res Treat..

[50] Hanson D, Murray PG, Coulson T, Sud A, Omokanye A, Stratta E (2012). Mutations in CUL7, OBSL1 and CCDC8 in 3-M syndrome lead to disordered growth factor signalling. J Mol Endocrinol..

[51] Hanson D, Murray PG, Sud A, Temtamy SA, Aglan M, Superti-Furga A (2009). The primordial growth disorder 3-M syndrome connects ubiquitination to the cytoskeletal adaptor OBSL1. Am J Hum Genet..

[52] Baron J, Savendahl L, De Luca F, Dauber A, Phillip M, Wit JM (2015). Short and tall stature: a new paradigm emerges. Nat Rev Endocrinol..

[53] Database of Single Nucleotide Polymorphisms (dbSNP). Bethesda: National Center for Biotechnology Information, National Library of Medicine. http://www.ncbi.nlm.nih.gov/SNP/. Accessed 1 July 2017.

[54] Diorio C, Brisson J, Berube S, Pollak M (2008). Genetic polymorphisms involved in insulin-like growth factor (IGF) pathway in relation to mammographic breast density and IGF levels. Cancer Epidemiol Biomarkers Prev..

[55] Verheus M, McKay JD, Kaaks R, Canzian F, Biessy C, Johansson M (2008). Common genetic variation in the IGF-1 gene, serum IGF-I levels and breast density. Breast Cancer Res Treat..

[56] Boyd N, Martin L, Stone J, Little L, Minkin S, Yaffe M (2002). A longitudinal study of the effects of menopause on mammographic features. Cancer Epidemiol Biomarkers Prev..

[57] Bremnes Y, Ursin G, Bjurstam N, Rinaldi S, Kaaks R, Gram IT (2007). Insulin-like growth factor and mammographic density in postmenopausal Norwegian women. Cancer Epidemiol Biomarkers Prev..

[58] Ruan W, Catanese V, Wieczorek R, Feldman M, Kleinberg DL (1995). Estradiol enhances the stimulatory effect of insulin-like growth factor-I (IGF-I) on mammary development and growth hormone-induced IGF-I messenger ribonucleic acid. Endocrinology..

[59] Ruan W, Monaco ME, Kleinberg DL (2005). Progesterone stimulates mammary gland ductal morphogenesis by synergizing with and enhancing insulin-like growth factor-I action. Endocrinology..

[60] Singh B, Smith JA, Axelrod DM, Ameri P, Levitt H, Danoff A (2014). Insulin-like growth factor-I inhibition with pasireotide decreases cell proliferation and increases apoptosis in pre-malignant lesions of the breast: a phase 1 proof of principle trial. Breast Cancer Res..

[61] Campagnoli C, Abba C, Ambroggio S, Peris C (2003). Differential effects of progestins on the circulating IGF-I system. Maturitas..

[62] Heald A, Selby PL, White A, Gibson JM (2000). Progestins abrogate estrogen-induced changes in the insulin-like growth factor axis. Am J Obstet Gynecol..

[63] Russo J, Moral R, Balogh GA, Mailo D, Russo IH (2005). The protective role of pregnancy in breast cancer. Breast Cancer Res..

[64] Haakensen VD, Lingjaerde OC, Luders T, Riis M, Prat A, Troester MA (2011). Gene expression profiles of breast biopsies from healthy women identify a group with claudin-low features. BMC Med Genomics..

