# Supplementary material for: Growth factor genes and change in mammographic density after stopping combined hormone therapy in the California Teachers Study
Source: BMC Cancer. 2018 Nov 6;18:1072. doi: 10.1186/s12885-018-4981-6 (PMC6220514; doi:10.1186/s12885-018-4981-6)
Supplement: Supplementary file 1 — Table S1. Comparison of characteristics of women who were included in the analyses with characteristics of participants who were included in the longitudinal set (i.e. both on-EPT and off-EPT mammograms were available) but excluded from the analyses. (DOCX 14 kb) [file 12885_2018_4981_MOESM1_ESM.docx]

Supplementary Table 1. Comparison of characteristics of women who were included in the analyses with characteristics of participants who were included in the longitudinal set (i.e. both on-EPT and off-EPT mammograms were available) but excluded from the analyses (Part of this table, the characteristics of the 284 women included in the current analysis, was reproduced with permission from Lee et al. 2014 originally published in Breast Cancer Research by BioMed Central. [12])

| Characteristics | Participants included in the current analysis  (non-Hispanic white EPT quitters)  (n=284) | Participants with both on-EPT and off-HT mammograms who were excluded in the current analysis  (n=138) | P-values |
| --- | --- | --- | --- |
| **At cohort enrollment** |  |  |  |
| Non-Hispanic white (N (%)) | 284 (100%) | 103 (75%) | - |
| Age (mean ± SD) | 50.5 ± 3.8 (range 41-60) | 50.1 ± 4.1 (range 41-59) | 0.34 |
| BMI (kg/m^2^) | 24.7 ± 4.9 (range 17-47) | 24.1 ± 5.1 (range 18-48) | 0.22 |
| Nulliparous women (N (%)) | 63 (22%) | 31 (22%) | 0.98 |
| Menopausal status |  |  | 0.85 |
| Premenopausal | 155 (55%) | 79 (57%) |  |
| Perimenopausal | 55 (19%) | 17 (12%) |  |
| Postmenopausal | 74 (26%) | 42 (30%) |  |
| Ever had breast biopsy (N (%)) | 40 (14%) | 20 (14%) | 0.91 |
| Positive 1st degree family history of breast cancer (N (%)) | 37 (13%) | 12 (9%) | 0.20 |
| **At mammogram substudy enrollment** |  |  |  |
| Age at interview* | 62.3 ± 3.6 (range 53-72) | 61.9 ± 3.9 (range 53-70) | 0.40 |
| BMI (kg/m^2^) | 25.7 ± 5.0 (range 18-47) | 25.7 ± 5.5 (range 16-48) | 0.92 |
| Positive 1st degree family history of breast cancer (N (%)) | 51 (18%) | 22 (16%) | 0.67 |
| N of mammograms in the past 10 years | 8.9 ± 2.2 (range 2-18) | 9.2 ± 2.5 (range 3-20) | 0.27 |
| **At time of mammography evaluated for density** |  |  |  |
| Age at time of mammogram while taking EPT | 56.4 ± 4.0 (range 45-67) | 56.0 ± 4.2 (range 47-67) | 0.34 |
| Age at time of mammogram while off HT | 60.1 ± 4.0 (range 49-71) | 57.2 ± 5.9 (range 43-69) | <0.0001 |
| BMI at time of mammogram while taking EPT | 25.7 ± 5.2 (range 18-47) | 25.3 ± 5.8 (range 17-43) | 0.63 |
| BMI at time of mammogram while off HT | 25.8 ± 5.0 (range 18-48) | 25.4 ± 6.0 (range 17-44) | 0.64 |
| Time interval between two mammograms (years) |  |  | <0.0001 |
| Off-HT mammogram before on-EPT mammogram | 0 (0%) | 51 (37%) |  |
| On-EPT mammogram before off_HT mammogram |  |  |  |
| ≤3 years | 141 (50%) | 35 (25%) |  |
| 4-5 years | 106 (37%) | 34 (25%) |  |
| 6-9 years | 37 (13%) | 18 (13%) |  |

Abbreviation: CTS, California Teachers Study; EPT, estrogen and progestin combined therapy; BMI, body mass index; HT, hormone therapy
